# Protein modification by biotinylated amoxicillin: usefulness in studies on allergy towards beta-lactams

**DOI:** 10.1186/2045-7022-4-S3-P37

**Published:** 2014-07-18

**Authors:** Adriana Ariza, Daniel Collado, Yolanda Vida, María Isabel Montañez, Ezequiel Pérez-Inestrosa, Miguel Blanca, María José Torres, Javier Cañada, Dolores Pérez-Sala

**Affiliations:** 1 IBIMA, Regional University Hospital of Malaga, UMA, Research Laboratory, Spain; 2 BIONAND-Andalusian Centre for Nanomedicine and Biotechnology and University of Malaga, Department of Organic Chemistry, Spain; 3 IBIMA, Regional University Hospital of Malaga, UMA, Allergy Unit, Spain; 4 Centro de Investigaciones Biológicas, Consejo Superior de Investigaciones Científicas, Department of Chemical and Physical Biology, Spain

## Background

Beta-lactam (BLs) antibiotics are among the drugs most frequently eliciting allergic reactions, thus posing an important clinical problem. Protein haptenation plays a key role in immunological reactions to BLs and this process is considered necessary for the activation of the immune system. Therefore, it is of interest to develop tools to monitor this process. We have synthesized a biotinylated amoxicillin derivative (AX-B) to be used in the characterization of protein haptenation by amoxicillin (AX).

## Methods

AX-B was obtained binding a biotin moiety at the lateral chain of AX. The recognition of AX-B by IgE of allergic patients was analyzed by RAST inhibition. AX and AX-B were used for the in vitro modification of proteins in serum and in cell culture. Modified proteins were separated by SDS-PAGE and detected by Western blot using an anti-AX antibody (for AX-modification) or HRP-streptavidin (for AX-B-modification). Distribution of intracellular protein adducts with AX-B was analyzed by confocal microscopy.

## Results

AX-B showed a chemical reactivity similar to AX and competition studies suggested that AX-B and AX can modify the same targets. AX and AX-B behaved similarly in RAST inhibition studies with sera of patients with non-selective allergy to BLs, whereas competition by AX-B was poorer with sera of AX-selective patients. AX-B allowed the detection of protein adducts with high sensitivity. This derivative bound to all of the previously identified major AX targets in serum but made possible to detect some new targets. In addition, in cell culture, AX-B protein adducts could be detected intracellularly.

## Conclusions

AX-B may constitute a valuable tool for the identification of AX targets with high sensitivity. The identity of novel AX-B targets will be the subject of future studies.

